# Prevention of striae gravidarum: study protocol for a pilot randomised controlled trial

**DOI:** 10.1186/s13063-018-2898-7

**Published:** 2018-10-12

**Authors:** Miriam Brennan, Mike Clarke, John Newell, Declan Devane

**Affiliations:** 1 School of Nursing & Midwifery, Aras Moyola, National University of Ireland Galway, Galway, H91 TK33 Ireland; 2 Centre for Public Health, School of Medicine, Dentistry and Biomedical Sciences, Institute for Health Sciences, Queen’s University Belfast, Belfast, BT12 6BA Northern Ireland; 3 School of Mathematics, Statistics, and Applied Mathematics, National University of Ireland Galway, Galway, H91 TK33 Ireland

**Keywords:** Striae gravidarum, Stretch marks, Protocol, Pilot trial, Randomised controlled trial

## Abstract

**Background:**

Striae gravidarum (stretch marks) are considered the most common connective tissue/skin change in pregnancy. Though not a health issue they can affect women in different ways, for example, cause stress or be an aesthetic or cosmetic concern. Many women use one or more of the commercially available products to try and prevent their development during pregnancy despite the fact that that there is a lack of high-quality evidence to support their use. There is a dearth of studies on the prevention of striae gravidarum and large, robust trials are lacking. Until such time as more products are investigated, much of the knowledge remains anecdotal. This pilot study will evaluate the feasibility of conducting a study to evaluate the effectiveness of a commercially available moisturising oil compared to no treatment for the prevention and reduction in severity of striae gravidarum.

**Methods:**

The definitive study will be a randomised controlled trial to evaluate the effectiveness of a moisturising oil (commercially available moisturising oil) compared to no treatment for the prevention and reduction in severity of striae gravidarum. This protocol is for a pilot randomised trial to evaluate the feasibility of conducting such a study. The pilot study will be a two-arm, unblinded, pragmatic parallel randomised trial with a 1:1 randomisation ratio between control and intervention groups. Women in the intervention group will be asked to apply a moisturising oil to their abdomen during pregnancy, while women in the control group will not use any treatment. It is proposed to recruit 20 primigravida, who are 12–16 weeks pregnant from an Irish Maternity Hospital, in each arm to assess the feasibility of running such a trial.

**Discussion:**

This pilot trial will evaluate the feasibility of conducting the main study to evaluate the effectiveness of a moisturising oil (commercially available moisturising oil) compared to no treatment for the prevention and reduction in severity of striae gravidarum. It will potentially initiate the generation of high-quality evidence to guide women in their choice of anti-stretch mark product.

**Trial registration:**

ISRCTN Registry, ISRCTN76992326. Registered on 14 July 2017.

**Electronic supplementary material:**

The online version of this article (10.1186/s13063-018-2898-7) contains supplementary material, which is available to authorized users.

## Background

Striae gravidarum or stretch marks occurring in pregnancy are considered the most common connective tissue change in pregnancy [1]. They affect all racial groups [2], with reported rates of occurrence usually in the range of 52–90% in women of different ethnicities [3–7]. Striae gravidarum are common during the first pregnancy [8] and usually present during the third trimester [9]. Striae start as ‘reddish slightly depressed streaks’ [9] (p. 111) and fade gradually [8] to leave pale wrinkled lines [10], which are permanent skin changes [11, 12] . These benign skin changes [4] occur commonly on the abdomen but are also seen on the breasts and thighs [8, 9, 13] and the hips and buttocks [14].

The exact cause of striae gravidarum remains unclear [5] but is considered to be related to the effects of stress on the tissue or stretching of the skin and hormonal effects [1, 15].

Risk factors associated with the development of striae gravidarum have been identified [4, 6], albeit inconsistently [16]. While some risk factors are modifiable, others are not [16]. Two of the commonly identified risk factors are higher weight gains in pregnancy [4, 5, 7] and higher birth weight babies [2, 4, 6, 13, 17]. Other risk factors include a family history [6, 7, 16, 18–20], a personal history of striae [18] and young maternal age [5, 13, 17, 19–21].

Though not a health issue [8, 14, 17], striae gravidarum have concerned women since early times [8] and continue to do so. They have caused anxiety or psychological/emotional distress to some women [8, 20, 22], while for others they are an aesthetic or cosmetic concern [4–6, 14, 18–20] and can impact negatively on a woman’s self-esteem and body image [14]. Consequently, many women during pregnancy have tried to prevent or treat striae gravidarum over the years and often at great expense [8] and indeed continue to do so [23].

### Prevention and treatment of striae gravidarum

There are many products available for women to choose during pregnancy purporting to prevent [24] or treat [14] striae gravidarum [5, 17, 21, 23, 25]. In one recent large survey involving 753 pregnant women [23], most respondents (78.2%, *n* = 589) indicated that they used a product to prevent or reduce the development of stretch marks during the current pregnancy and over one-third (36.5%, *n* = 210) had used two or more products.

The highest-quality evidence available to date on the prevention of striae gravidarum is from a recent Cochrane Review [26] which evaluated the effects of topical preparations on the prevention of stretch marks in pregnancy. Six trials with 800 women were included in the review which found that there was no statistically significant average difference in the development of striae gravidarum in women who received topical preparations with active ingredients compared to women who received a placebo or no treatment (average risk ratio [RR] 0.74; 95% confidence interval [CI] 0.53–1.03; five trials, 474 women; random-effects model, Tau^2^ = 0.09, I^2^ = 65%). There was also no statistically significant average mean difference in the severity of striae gravidarum (standardised mean difference [SMD] – 0.31; 95% CI – 1.06–0.44; two trials, 255 women; Tau^2^ = 0.26, I^2^ = 87%).

Similarly, there was no statistically significant difference between women who received topical preparations with active ingredients compared to women who received other topical preparations with active ingredients in the development of striae gravidarum (average RR 0.51; 95% CI 0.16–1.60; two trials, 305 women; Tau^2^ = 0.53, I^2^ = 74%) or in the severity of striae gravidarum (mean difference [MD] – 0.20; 95% CI – 0.53–0.13; one trial, 206 women; heterogeneity not applicable). The authors concluded that they found no high-quality evidence to support the use of any of the topical preparations evaluated in the review [26].

### Why a trial is needed?

The prevention of striae is important to women [27] and women often ask how they can be prevented during pregnancy [5, 16]. There is a dearth of studies on their prevention and large, robust trials are lacking [26, 28]. This protocol is for a pilot randomised trial to evaluate the feasibility of conducting a larger trial and will compare a moisturising oil versus no treatment for the prevention and reduction in the severity of striae gravidarum. The null hypothesis for the future definitive trial is that there will be no difference in the proportion of participants who develop striae gravidarum or in the severity of the striae between the group who apply the moisturising oil and the group who do not apply any product.

All aspects of the pilot protocol presented here are informed by the 2013 Standard Protocol Items as per Recommendations for Interventional Trials (SPIRIT) statement [29], which seeks to ensure high-quality trial protocols.

## Methods

**Fig. 1:**
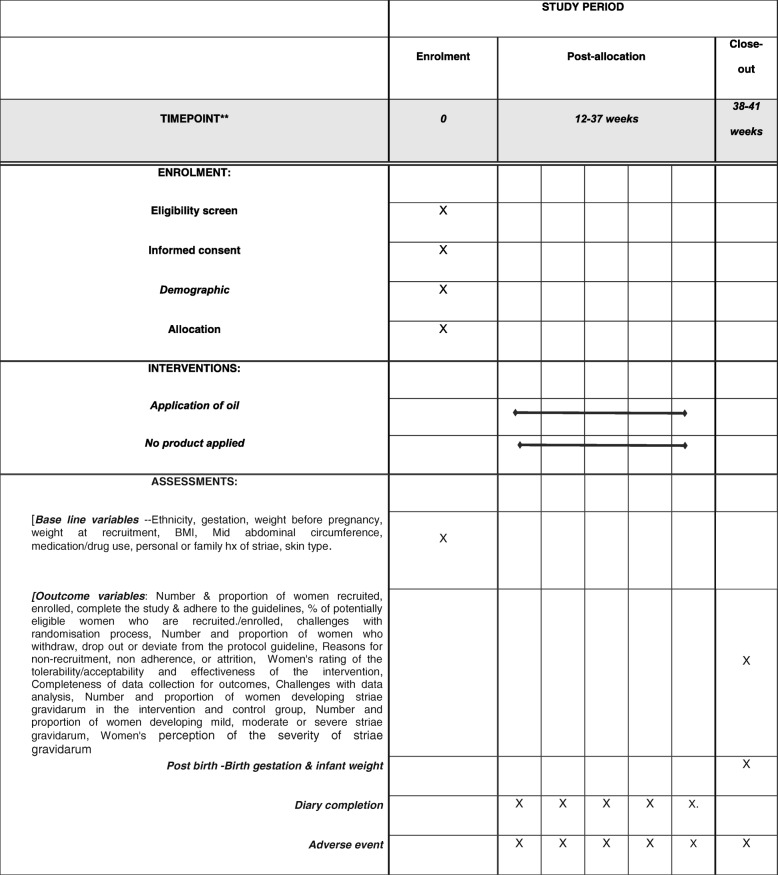
Standard Protocol Items: Recommendations for Interventional Trials (SPIRIT) schedule of enrolment, interventions and assessments for the duration of the study

The definitive study will be a randomised controlled trial to evaluate the effectiveness of a moisturising oil (commercially available moisturising oil) compared to no treatment for the prevention and reduction in severity of striae gravidarum. This protocol is for a pilot randomised trial to evaluate the feasibility of conducting such a study. The study will follow the SPIRIT guidelines for the design and conduct of a trial (Fig. 1; Additional file 1). The pilot study will be a two-arm, unblinded, pragmatic parallel randomised trial with a 1:1 randomisation ratio between control and intervention groups, comparing the effectiveness of a moisturising oil (commercially available moisturising oil) compared to no treatment for the prevention and reduction in severity of striae gravidarum.

While a randomised trial design is the most effective design for testing the effects of a healthcare intervention [30], a pilot trial enables the testing of the feasibility and acceptability of undertaking a larger trial [31]. It will also contribute to modification of the trial protocol for a larger study [31].

### Objectives of the pilot trial

- Measure recruitment, enrolment, completion, adherence and attrition (withdrawal, drop outs) rates;
- Evaluate recruitment and randomisation processes;
- Evaluate the data collection instruments;
- Collect qualitative data on reasons for refusal to participate; reasons for non-adherence with the intervention and control guidelines and reasons for attrition;
- Ascertain women’s rating of the tolerability/acceptability and effectiveness of the intervention;
- Measure the completeness of data collection for outcomes;
- Evaluate the data analysis methods.

As this is a feasibility study, formal hypothesis testing will not be performed. Suitable descriptive statistics and graphical summaries will be used to summarise participant characteristics. Means and standard deviations will be used for continuous variables; counts and percentages will be used for categorical variables with accompanying 95% CI estimates.

### Participants

#### Eligibility criteria for participants

##### Inclusion criteria

- Primigravid women;
- Singleton pregnancy;
- 12–16 weeks of gestation at time of recruitment;
- Absence of abdominal striae;
- Aged ≥ 18 years at recruitment;
- English-speaking women.

##### Exclusion criteria

- Multigravid women (due to risk of having striae from an earlier pregnancy);
- Women taking corticosteroids for any reason (due to their association with skin striae);
- Multiple pregnancy (due to increased maternal weight gain and skin stretching);
- Women with a known hypersensitivity to agents in baby oil (due to risk of allergic reaction);
- Women with known disorders or illnesses that may be associated with striae, e.g. Marfan syndrome, Cushing syndrome;
- Women who decline to discontinue other creams or lotions;
- Women who are unable to give informed consent.

### Interventions

#### Experimental intervention

The intervention product for this pilot trial is a moisturising oil (commercially available moisturising oil) marketed to increase skin moisture. It contains an oil base: Paraffinum liquidum and Isopropyl Palmitate and parfum (Johnson’s baby oil).

Each woman randomised to the experimental intervention group will receive two plastic bottles of the moisturising oil with 200 mL in each. This is based on each woman applying ~ 2 mL per day (14 mL per week). Based on each woman applying the oil over 26 weeks, i.e. up until approx. 38 weeks of gestation (Fig. 1), each woman will require approximately 400 mL of oil in total. Each woman will be asked to apply ~ 2 mL to their abdominal skin daily after showering and before drying their skin. This will equate with two pumps of oil to ensure consistency in the amount applied.

Instructions for applying the oil will be printed on a label on the bottles as will the appropriate safety advice (see “Safety and undesirable effects” below). Women will be advised to apply the oil into their abdominal skin once daily from 12 weeks of gestation. They will be advised to apply the oil to their damp abdominal skin after showering and to ‘pad dry’ the area. The researcher will instruct them in the application of the oil. Women will be asked not to use any other skin product on their abdomen to maintain treatment fidelity and to complete a diary record of their daily application of the intervention oil and of any other product applied to their abdomen during the trial period (Fig. 1). They will also be asked to keep the empty oil bottles and bring them to the outcome assessment interview at 38–41 weeks.

#### Control intervention

Women allocated to the control group will not use any treatment and will be asked not to use any other skin product on their abdomen to maintain treatment fidelity. They will also be asked to keep a diary record (Fig. 1) and to document any product applied to their abdominal skin so that this can be assessed as part of the pilot trial.

All women will receive a two-weekly text to remind them about their participation in the trial and to complete the diary. A text message reminder will also be sent to women at 37 weeks to bring their empty oil bottles to the outcome assessment interview as appropriate.

#### Outcome measures

In order to evaluate the feasibility of conducting a definitive randomised controlled trial to evaluate the effectiveness of a moisturising oil (commercially available moisturising oil) compared to no treatment for the prevention and reduction in severity of striae gravidarum, the outcomes for this pilot study are:

1. Number and proportion of women who are recruited, enrolled, complete the study and adhere to the intervention and control guidelines;
2. Percentage of potentially eligible women who are recruited/enrolled to the study;
3. Challenges with the randomisation process;
4. Number and proportion of women who withdraw, drop out or deviate from the protocol guidelines;
5. Reasons for non-recruitment, non-adherence or attrition;
6. Women’s rating of the tolerability/acceptability and effectiveness of the intervention;
7. Completeness of data collection for outcomes;
8. Challenges with data analysis;
9. Number and proportion of women developing striae gravidarum in the intervention and control groups;
10. Number and proportion of women developing mild, moderate or severe striae gravidarum;
11. Women’s perception of the severity of striae gravidarum (Fig. 1).

In addition to the above outcomes, birth data will also be collected following each birth from the labour ward register on birth gestation and infant weight (Fig. 1) and recorded on the outcomes assessment form.

#### Assessment of the development and severity of striae gravidarum

The development and severity of striae will be assessed using the Davey instrument [32] and the classification system of mild, moderate or severe as used by Soltanipour et al. [33]. It will entail dividing the abdomen into four quadrants and using an ordinal scoring scale to score each quadrant based on the number of striae present as above. However, the numbers in each quadrant will be interpreted as follows: 0 = no striae in a quadrant; 1 = striae do not affect a quadrant completely; and 2 = striae affect a quadrant completely [33]. Scores for each quadrant will be combined and graded as follows: women with a total of ‘0’ will be graded as having ‘none’, ‘1–3 mild striae, 4–6 as moderate striae, and 7–8 as severe striae’ [33]. The assessment will take place in a well-lit room with adequate natural day or artificial lighting and will be undertaken by the researcher or a research assistant.

#### Sample size and feasibility of sample size

A sample size of 228 women (114 per arm) is needed in the definitive trial to have 80% power to detect an absolute difference in the proportion of participants developing stretch marks of 0.20 [14] that is 0.47 [23] to 0.27 at the 5% significance level based on a two-sample test for a binomial proportion. This sample allows for 20% attrition. However, for the pilot trial it is proposed to recruit 20 women in each arm to assess the feasibility of running such a trial and to inform variable parameters. This sample size equates to 17.5% of the sample size needed for the main trial. The pilot data will be incorporated into the definitive trial, if feasible, as a seamless trial with an indicator variable used to distinguish pilot from non-pilot data. Furthermore, the attrition rate in the pilot trial will inform the final sample size for the main trial. This sample size is considered acceptable for a pilot study [34].

The pilot trial will involve one large maternity hospital with approximately 3000 births annually, which includes approximately 1100 first-time mothers. It is anticipated that circa 80 mothers will be booked per month and it will be feasible to recruit 40 first-time mothers over a three- to four-month period. This takes into consideration women who will refuse to participate and women who will not be eligible.

### Randomisation

#### Random sequence generation

The random allocation sequence will be generated using the random number generator (the Mersenne Twister) available in StatsDirect [35] by an independent person. Women will be allocated to the intervention and control group using a 1:1 randomisation ratio using random block sizes to ensure that the group to which each woman will be allocated will be determined by a chance process and cannot be predicted.

#### Allocation concealment mechanism

Allocation concealment will be ensured using sequentially numbered opaque envelopes prepared previously by a person independent from recruitment or allocation of participants to groups. Integrity of the process will be ensured by the researcher recording the woman’s name and the number of the next unopened envelope in the woman’s trial register form in the presence of another colleague and by both persons signing the unopened envelope. Following this, the envelope will be opened and the group allocation and study ID revealed [36]. Group allocation and study ID number which corresponds to the envelope number for that woman will be recorded on the trial register form.

### Blinding

#### Blinding of participants and personnel (performance bias)

Due to the technical design of the intervention, the women, the healthcare professionals caring for them and the researcher will not be blinded to which group the participant is randomised.

#### Blinding of outcomes assessment (detection bias)

As above, the outcome assessor will not be blinded to the group to which the participant is randomised.

#### Recruitment to the trial

Given that most first-time mothers will be eligible for inclusion in the study, an ethics committee-approved study information pack will accompany the booking appointment notification sent to each first-time mother by the clinic administrator. The study pack will include a letter inviting the woman to participate in the study and an information leaflet describing the background, purpose and study details. The information leaflet will provide contact details should potential participants have questions about any aspect of the study. This process ensures participants have time to decide whether they wish to participate and complies with best practice in obtaining consent [37].

The researcher will approach women as they wait for their booking appointment in the clinic while the support of midwives and obstetricians will be sought in identifying potentially eligible women. Thus, at each antenatal booking clinic during the recruitment phase, all primigravid women will be approached by the researcher or a research assistant and given the opportunity to discuss the study details before inviting them to participate in the study. A trial register form will be completed for each woman and each woman screened will be assigned a unique study ID number.

#### Enrolment to the trial

**Fig. 2:**
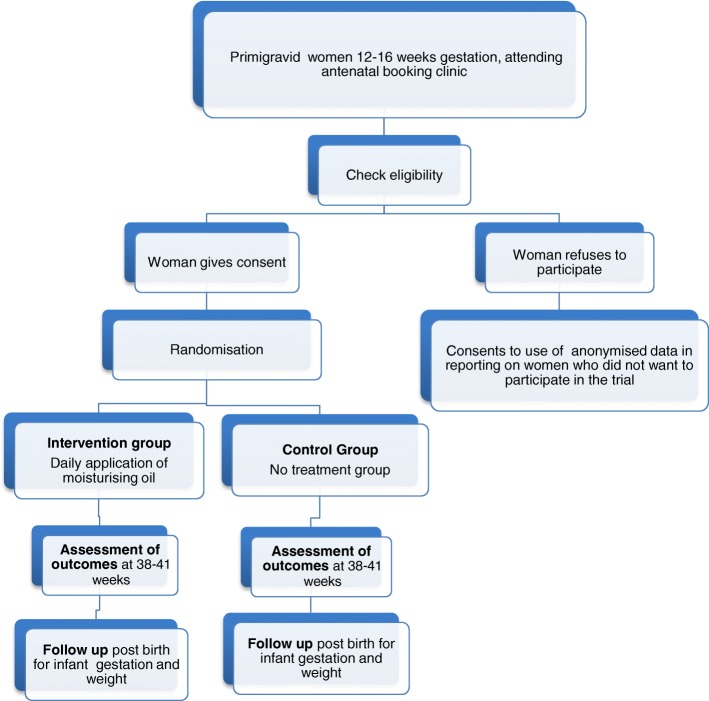
Flow of participants through the trial

Women who are attending for their antenatal booking appointment and who are screened and deemed eligible and agree to join the study will be asked by the researcher or a research assistant to give written consent before randomisation (Fig. 2), including consent to receiving two-weekly reminder texts, which reminds participants to complete the daily study diary.

Women who agree to participate will be asked to sign three copies of the consent form and these will also be signed by the researcher. One copy will be returned to the woman, one will be put in the woman’s hospital records and the third will be kept for the research records. Women who are eligible and who decline to participate will be asked for their consent to disclose their reasons for non-participation. Further, women who subsequently decide to withdraw from the study will be asked to contact the researcher as outlined on the participant information leaflet**.** Women will then be asked by the researcher over the phone to give their consent to: (1) retention of data collected before their withdrawal from the study; or (2) retention of data collected before their withdrawal from the study and the inclusion of data collected routinely; or (3) that they wish to withdraw completely.

When written consent is obtained as outlined, the researcher will select the next sequentially numbered opaque envelope with the woman’s allocation. The researcher will then record the woman’s name and the envelope number in the woman’s trial register form in the presence of another colleague; they will both then sign the unopened envelope. Following this, the researcher will open the envelope revealing the group allocation and study ID, both of which will be recorded in the trial register form as above.

Women will complete a demographic and baseline characteristics’ data form as part of the case report form (CRF), which seeks information on name, date of birth, medical history, drug use, smoking status, maternal body mass index (BMI) at enrolment (BMI will also be assessed at 38–41 weeks of gestation at time of outcome assessment) and family history of first-degree relative developing striae in pregnancy.

### Participant follow-up

Women will be followed up over a period of approximately 26 weeks, during which two-weekly reminder texts to complete the study diary will be sent. Outcome data will be collected at the antenatal clinic appointment at 38–41 weeks of gestation (Fig. 1).

Based on the clinic attendance lists, the researcher will arrange to meet each woman at one of their antenatal appointments at 38–41 weeks of gestation as above to undertake the outcome assessment. Women will be assessed for the development of striae and the severity of striae using the assessment instruments outlined above. In addition, the following data will be collected:

- Gestation at assessment;
- Current weight (to determine weight gain [% and absolute gain] during pregnancy);
- BMI;
- Mid-abdominal circumference;
- Development of polyhydramnios;
- Gestation at onset of striae as applicable;
- Development of striae in other body areas;
- Adherence to the intervention or control guidelines or not and reasons for same;
- Adverse event/undesirable effect (see below);
- Women’s evaluation of the tolerability and the effectiveness of the intervention;
- Women’s perceptions of the severity of striae gravidarum.

In addition, following the birth, the researcher will collect data on the following, from the labour ward register:

- Gestational week at birth;
- Weight of infant (Fig. 1).

### Safety and undesirable effects

In keeping with the safety advice for the moisturising oil, women will be advised at recruitment that the oil is for external use only and should be kept out of the reach of children. They will be advised that in the event of a rash occurring or breathing problems or any other undesirable effect associated with the oil, that they discontinue the application of the oil and contact their general practitioner. They will also be asked to contact the researcher who will complete an undesirable effect form and the undesirable effect record log**.** The researcher will also complete a Cosmetic Product Undesirable Effect Report for the Health Products Regulatory Authority (HPRA) [38].

Adverse events for this study are classified as undesirable effects and serious undesirable effects [38] as follows:

- Undesirable effect – ‘…an adverse reaction to a cosmetic product under normal or foreseeable conditions of use’, (e.g. ‘…irritant and allergic effects, sensitivity to light and itching’;
- Serious undesirable effect – ‘…an undesirable effect which results in temporary or permanent functional incapacity, disability, hospitalization, congenital anomalies, an immediate vital risk or death’.

### Criteria for proceeding to the main trial

The decision to proceed to the main trial will be made by the Trial Steering Committee based on the following criteria:

- ≥ 40% of eligible women can be recruited;
- ≥ 70% of women adhere to the intervention and control guidelines;
- <  20% of women lost to follow-up.

### Data management

All data will be recorded carefully on the appropriate CRFs, which will be stored in a locked cabinet in the researcher’s work office. Quality of CRF completion will be monitored on an ongoing basis by the researcher. Only the researcher and a research assistant will have access to the paper data, while the researcher, her supervisor, a research assistant and a statistician will have access to the electronic data, which will be anonymised based on individual participant trial numbers and stored in a password-protected file on a password-protected, encrypted laptop. All data will be kept secure by the researcher for a period of five years and then shredded.

### Statistical analysis

Data will be entered into a secure database using the Statistical Packages for the Social Sciences (SPSS) version 21 [39] as collected. Data will be coded and cleaned. As this is a feasibility study, formal hypothesis testing will not be performed. Suitable descriptive statistics and graphical summaries will be used to summarise participant characteristics. Means and standard deviations will be used for continuous variables; counts and percentages will be used for categorical variables with accompanying 95% CI estimates.

In addition, numbers and percentages of women recruited, eligible, randomised, enrolled and who completed the trial will be outlined in a CONSORT flow chart.

### Intervention fidelity

Complete intervention adherence will be defined as the woman applying the allocated oil daily over approximately 26 weeks. Anything less than this will be considered incomplete adherence, with < 10 applications in total over the approximately 26 weeks being regarded as non-adherence.

### Trial monitoring

#### Data monitoring

The researcher will monitor recruitment and reasons for non-recruitment of potentially eligible women as the trial proceeds. The researcher will ensure that sufficient time is given for individual discussion about the trial to potential participants. Due to the aim, objectives and size and of this pilot study, it was not considered necessary to have a data monitoring committee.

#### Trial management

The day-to-day management of the trial is the researcher’s responsibility under the supervision of her supervisors, while a Steering Group will also be established to supervise the trial as below.

#### Trial Steering Committee (TSC)

A TSC will be established to provide overall supervision of the trial and to ensure that the trial is conducted rigorously. It will include a midwifery manager and a research expert. The TSC will discuss protocol once a month and at the end of the trial. Further, they will offer guidance on continuation, modification or cessation of the trial as appropriate and will review any cases of reported trial-related undesirable effects.

## Ethics

The trial will be conducted in accordance with the WMA Declaration of Helsinki ethical principles for research [40]. Ethical approval was granted by the local Clinical Research Ethics committee.

In the event of the protocol requiring any amendments, the researcher will inform the ethics committee, the participants and any other party as appropriate. Consent will be obtained by the researcher as outlined above. All study data will be kept confidential as above before, during and following the study. Access to the dataset will be restricted to the persons named above.

## Discussion

Many women use the commercially available anti-striae products to try and prevent the development of striae gravidarum. This pilot trial will evaluate the feasibility of conducting the main study to evaluate the effectiveness of a moisturising oil (commercially available moisturising oil) compared to no treatment for the prevention and reduction in severity of striae gravidarum. It will potentially initiate the generation of high-quality evidence to guide women in their choice of anti-stretch mark product which heretofore is unavailable.

## Trial status

Protocol version: 2–25 January 2018.

Date recruitment began: 18 July 2017 for two weeks, recommenced February 2018; however, no participant has been enrolled to date.

Approximate date recruitment will be completed: May 2018.

## Additional file

Additional file 1: SPIRIT 2013 Checklist: Recommended items to address in a clinical trial protocol and related documents. (DOC 122 kb)

## Abbreviations

IBM Corp: International Business Machines Corporation
WMA: World Medical Association
